# Methodological challenges in monitoring new treatments for rare diseases: lessons from the cryopyrin-associated periodic syndrome registry

**DOI:** 10.1186/1750-1172-8-139

**Published:** 2013-09-10

**Authors:** Hugh Tilson, Paola Primatesta, Dennis Kim, Barbara Rauer, Philip N Hawkins, Hal M Hoffman, Jasmin Kuemmerle-Deschner, Tom van der Poll, Ulrich A Walker

**Affiliations:** 1 UNC Gillings School of Global Public Health, Chapel Hill, NC, USA and Chair, CAPS Registry Steering Committee, Chapel Hill, NC, USA; 2 Novartis Pharma AG, Basel, Switzerland; 3 Novartis Pharmaceuticals Corporation, East Hanover, NJ, USA; 4 University College London Medical School, London, United Kingdom; 5 University of California at San Diego, San Diego, CA, USA; 6 University Hospital Tuebingen, Tuebingen, Germany; 7 Academic Medical Center, University of Amsterdam, Amsterdam, Netherlands; 8 Rheumatologische Universitäts-Poliklinik, Felix-Platter Spital, Basel, Switzerland

**Keywords:** Cryopyrin-associated periodic syndrome, Registry, Epidemiology, Observational study

## Abstract

**Background:**

The Cryopyrin-Associated Periodic Syndromes (CAPS) are a group of rare hereditary autoinflammatory diseases and encompass Familial Cold Autoinflammatory Syndrome (FCAS), Muckle-Wells Syndrome (MWS), and Neonatal Onset Multisystem Inflammatory Disease (NOMID). Canakinumab is a monoclonal antibody directed against IL-1 beta and approved for CAPS patients but requires post-approval monitoring due to low and short exposures during the licensing process. Creative approaches to observational methodology are needed, harnessing novel registry strategies to ensure Health Care Provider reporting and patient monitoring.

**Methods:**

A web-based registry was set up to collect information on long-term safety and effectiveness of canakinumab for CAPS.

**Results:**

Starting in November 2009, this registry enrolled 241 patients in 43 centers and 13 countries by December 31, 2012. One-third of the enrolled population was aged < 18; the overall population is evenly divided by gender. Enrolment is ongoing for children.

**Conclusions:**

Innovative therapies in orphan diseases require post-approval structures to enable in depth understanding of safety and natural history of disease. The rarity and distribution of such diseases and unpredictability of treatment require innovative methods for enrolment and follow-up. Broad international practice-based recruitment and web-based data collection are practical.

## Background

The treatment experiences of patients with rare diseases are difficult to track and monitor. Cryopyrin-Associated Periodic Syndromes (CAPS), specifically Familial Cold Autoinflammatory Syndrome (FCAS), Muckle-Wells Syndrome (MWS), and Neonatal Onset Multisystem Inflammatory Disease (NOMID), are a group of rare hereditary autoinflammatory diseases, with estimated population frequency ranging from 1–3 per million [1]. These syndromes are typically a result of an autosomal dominant or *de novo* mutation of the cold-induced auto-inflammatory syndrome 1 (CIAS1)/nod-like receptor protein 3 (NLRP3) gene on chromosome 1 [2]. Although it remains poorly understood precisely how CIAS/NLRP-3 mutations cause inflammatory diseases, it is known that the protein encoded by this gene, NALP3 or cryopyrin, interacts with other intracellular proteins to form an intracellular complex called the inflammasome, resulting in an overproduction of active interleukin 1 (IL-1) beta, a proinflammatory cytokine [2,3].

CAPS generally manifest as life-long episodes of recurrent fever accompanied by differing degrees of neutrophil-mediated systemic inflammation. They are now regarded as a spectrum of overlapping traits and differences in severity, rather than distinct genetic disorders [4].

FCAS and MWS, on the less severe end of the spectrum, are typically first noted in infancy, early childhood or adolescence; while NOMID, also known as Chronic Infantile Neurologic Cutaneous Articular (CINCA) syndrome is a severe, sporadic form of the condition presenting in the neonatal period with multi-organ system inflammatory involvement, including significant central nervous system manifestations, not seen in other forms of CAPS.

Knowledge of the disease, although improving, is still limited. Disease symptoms generally appear in early childhood, but sensorineural deafness, one characteristic feature of MWS, develops in up to two-thirds of patients in later childhood and progresses through adulthood. Systemic amyloidosis develops in up to 25% of MWS patients and often leads to renal failure in adulthood [5]. The severity of NOMID is variable, and death may occur in young adulthood in 20% of the patients because of infection, secondary amyloidosis, or cachexia [6]. Clinical experience is based on few specialists and centres in any country, each caring for a very limited number of patients. Various symptomatic treatments are used to alleviate the pain and discomfort associated with the inflammatory flares, with limited success. Many patients are prescribed corticosteroids, which, although in high doses can reduce symptoms, cannot be used long-term because of side effects. With the identification of the genetic basis for the disease and the common pathway of IL-1 beta activation, new approaches to treat these conditions have been identified. Canakinumab, unlike other IL-1 inhibitor agents (e.g. anakinra or rilonacept), specifically blocks only IL-1 beta, the form of the IL-1 that mediates disease flares in these auto-inflammatory diseases. The efficacy and safety profile of canakinumab was demonstrated in the clinical trials carried out during the development program. Though exact prevalence is unknown, based on an estimate of one case per million people, canakinumab has been used for treatment of >65% of the target population [unpublished internal data]. As with all very rare (orphan) diseases, the clinical trials included a very limited number of patients treated under very controlled circumstances. The original drug approval dossier included data on a total of 78 CAPS patients, including 9 FCAS, 63 MWS, 5 MWS/NOMID and 1 NOMID patient with an overall exposure of 69 patient-years and a treatment duration of up to 3½ years; therefore, the post-approval period was considered a critical phase to gather more knowledge regarding the short- and long-term safety, effectiveness and treatment patterns associated with the use of the product.

To shed further light on the natural history of the disease and to observe the beneficial and adverse effects of the treatment under real-life circumstances, an international registry was implemented.

## Methods

Methods for this registry were developed in accordance with the STROBE guidelines [7] and the Registries User’s Guide [8]. The Registry is entirely observational. As such, no exclusion criteria were applied: all patients treated with canakinumab are eligible to be enrolled, irrespective of canakinumab label or other recommendations.

Likewise, as an observational study, no protocol–mandated visits or procedures were specified. Treatment and diagnostic decisions regarding the patient’s disease and care were to be determined by the physician according to standard of care and local clinical practice. Data collection was aligned with standard medical practice and captured during routine clinic visits instead of a lock-step periodic visit/form (no periodic follow-up or form was required). The Registry data therefore result from routine medical assessments performed during the initiation and follow-up of canakinumab treatment, and also depended on safety and other clinical outcomes. Data collected at baseline, i.e. at initiation of canakinumab therapy, included information which had otherwise already been collected: patient characteristics (including genotype and phenotype) and disease course, non-auto-inflammatory related medical history, laboratory examinations, and treatment with canakinumab and other medications. Treatment outcomes, including indicators of effectiveness and safety, and dosing of canakinumab treatment are being collected over the successive five years. In routine clinical care, most patients receive canakinumab approximately every 8 weeks at their physician’s office (recommended data collection schedule shown in Table 1); however it is recognized that in the clinical setting, dosing may be delayed or interrupted due to personal reasons, physical condition or co-morbidities.

**Table 1:** **CAPS registry** - **recommended data collection schedule**

**Period**	**Baseline**	Follow-up (until study end or premature discontinuation)*
**Timing**	**Enrollment**	**Every 6 months**
Informed consent	x	
CAPS phenotype	x	
CAPS genotype (NLRP-3 mutation), if available	x	
Non-CAPS medical history	x	
Vital signs (height, weight, blood pressure)	x	x
Ilaris dosing/status	x	x
Historical and concomitant medications for auto-inflammatory disease*	x	x
Selected AEs*		x
Other serious AEs and non-serious adverse reactions*		x
CAPS clinical assessment *	x	x
CAPS-related clinical testing results, if performed	x	x
Selected local laboratory testing	x	x
Sexual development status (paediatric only)	x	x
Neurocognitive status (paediatric only)	x	x
Cerebrospinal fluid analysis	x	x
Vaccination record and outcome	x	x
Pregnancy status (females of child-bearing potential only)		x
Registry disposition		x

* These assessments may also be conducted during follow-up for patients who discontinue canakinumab but remain in the Registry.

A Steering Committee, consisting of auto-inflammatory disease/CAPS specialists, an epidemiologist, an infectious disease specialist and a rheumatologist, was established to provide scientific guidance on the set-up, conduct, enrolment and analysis of the Registry. The Steering Committee, recognizing the unique challenges of a Registry for such rare conditions, also undertook the role of key informant and communicator.

An enrolment target of 200 patients was established (since the manuscript was submitted a protocol amendment revised the targeted sample to 260 patients); this choice aimed to double the numbers of patients and their experience, previously described in the published literature.

To achieve this number, sites that had participated in clinical trials during the development program and had experience in the disease were asked to participate and recommend further sites where treatment of numbers of CAPS patients adequate to justify training and recruitment was likely. Additional sites were identified in countries (e.g. Switzerland and Belgium) in which the participation of CAPS patients in the registry was a request of the local health authorities.

Ethical committee approval and if required, approval of local health authorities were obtained for all participating sites. Patients receiving canakinumab treatment could be enrolled in this registry irrespective of indication except in those countries, e.g. Spain, where only CAPS patients could be enrolled in accordance to local regulations. Informed consent and assent as appropriate under the national laws and practices in each country were signed by the participants or in case of minors by parents and child of adequate age.

**Figure 1:**
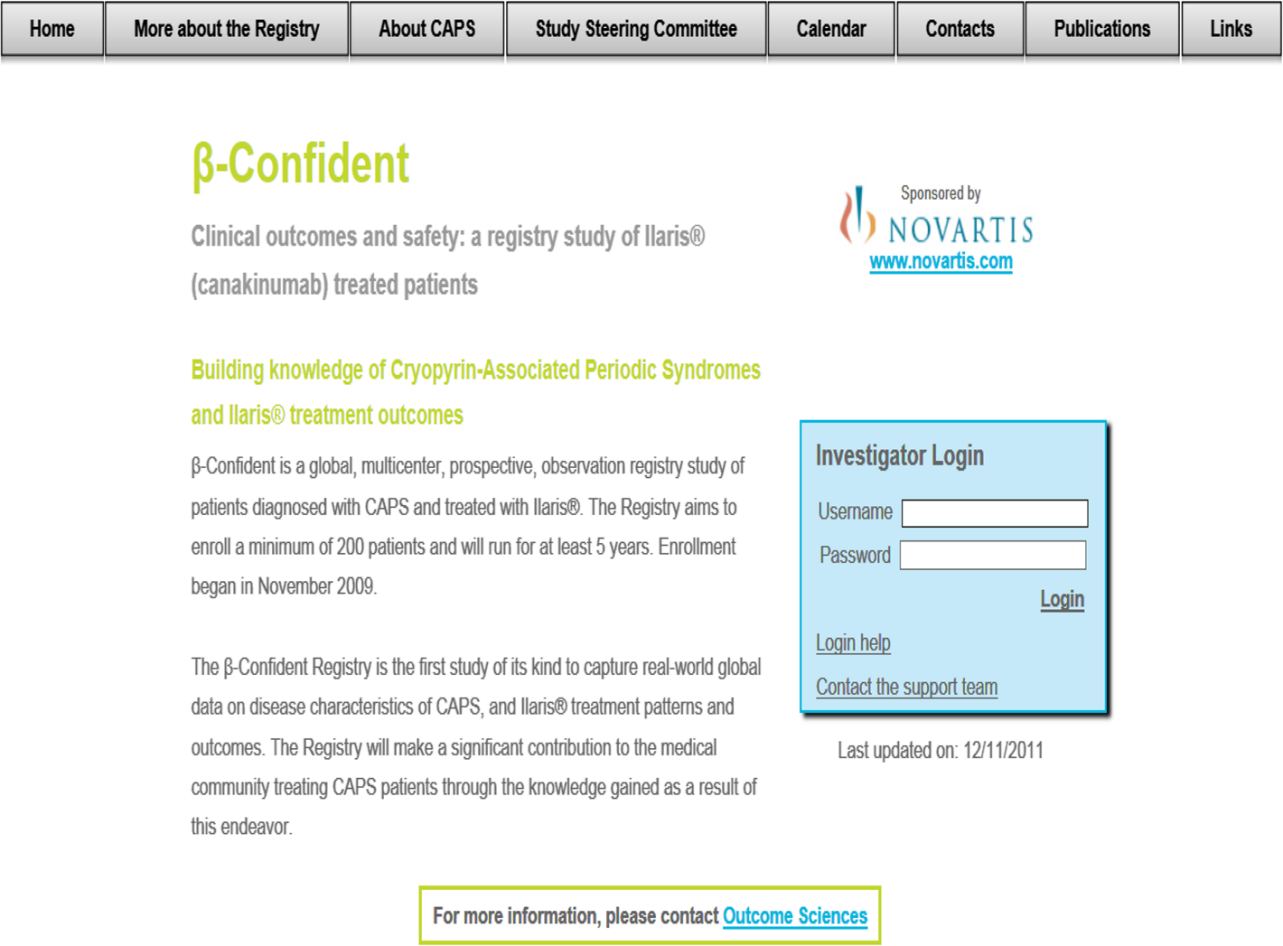
CAPS login web page.

To provide ease of data collection, expedite enrolment and facilitate follow-up, each center was provided training in a secure, password-protected, electronic data capture (EDC) system accessible through the internet (http://www.bconfidentregistry.com), as shown in Figure 1. Access to the EDC database has been given to all participating site personnel after they were trained on the system. Enrolment started in November 2009 and is ongoing for children. The follow-up of enrolled patients is ongoing and will continue for 1 year after enrolment of the last patient. This paper presents data entered in the Registry by Dec 31, 2012.

## Results

**Figure 2:**
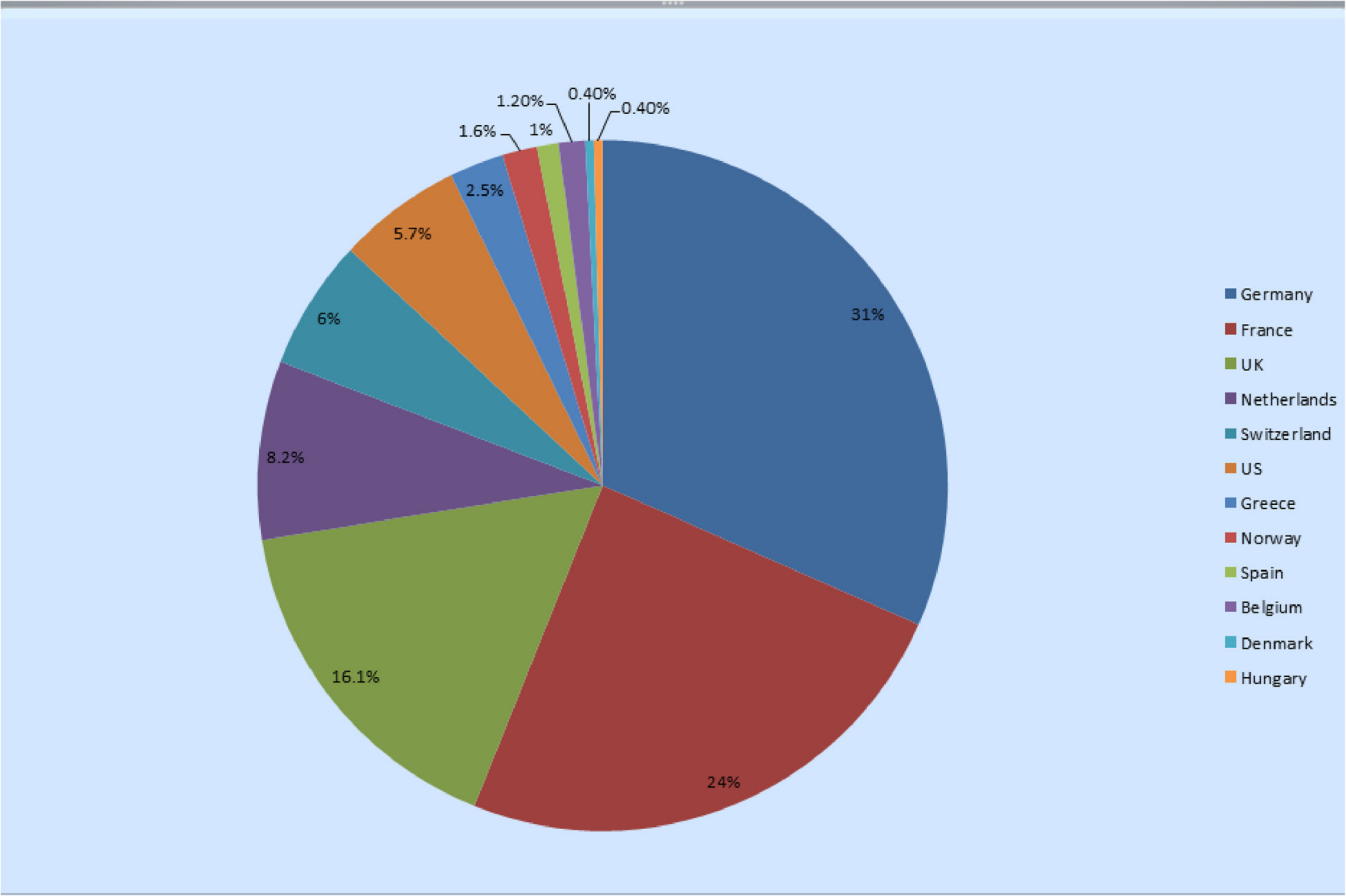
**Patient recruitment percentage per country.** The proportion of patients recruited from each enrolling country.

This recruitment strategy was successful at meeting the enrolment targets. By December 31, 2012, 241 patients from 43 centres in 13 countries were entered in the registry. Out of 43 participating sites, 23 sites were university hospitals, 5 were public hospitals, 5 private practices, and 1 was a private hospital (the information was not available from 9 sites). Enrolling countries are shown in Figure 2. Initial recruitment was higher in those centers that had participated in previous clinical trials: there were 17 such sites out of the 43 (39.5%), contributing overall with 76% of patients.

The distribution of patients at enrolment by condition, age and gender is shown in Table 2. Of all patients enrolled until December 2012, 80 (33%) were children (<18 years) at the time of enrolment (of whom 4 were <4 years of age); and 47.3% were males. The majority of patients was diagnosed with MWS (144, 59.8%), while 35 (15%) had a diagnosis of FCAS, 23 (9.5%) of NOMID and 31 had other diagnoses (including atypical CAPS, systemic juvenile idiopathic arthritis (SJIA), familial Mediterranean fever (FMF) and other autoinflammatory diseases). Information about the diagnosis is missing for 8 patients. For FCAS, the ratio of male:female was 2:1 while for the other conditions it was close to 1:1. To monitor the safety of treatment after approval, all patients treated with canakinumab could be enrolled in the CAPS registry independent of diagnosis. To date, 21 patients have permanently discontinued the study (i.e. they are no longer on canakinumab), including 3 who withdrew, 5 who were lost to follow-up, 2 who changed physician, 1 who died and 10 for other reasons.

**Table 2:** Demographics and baseline characteristics of patients* **enrolled** (**at Dec 31st**, **2012**)

**Variable n (%)**	**FCAS N = 35**	**MWS N = 144**	**NOMID N = 23**	Others** **N = 31**
**Gender**				
Male	12 (34.3)	74 (51.4)	11 (47.8)	15 (48.4)
Female	23 (65.7)	70 (48.6)	12 (52.2)	16 (51.6)
**Age**				
< 4 years	0	2 (1.4)	0	2 (6.5)
4 - <18 years	7 (20)	35 (24.3)	14 (60.9)	17 (54.8)
≥ 18 years	28 (80)	107 (74.3)	9 (39.1)	12 (38.7)

* Information about indication is missing for 8 patients.

** Others include atypical CAPS, SJIA, FMF and other autoinflammatory diseases.

**Figure 3:**
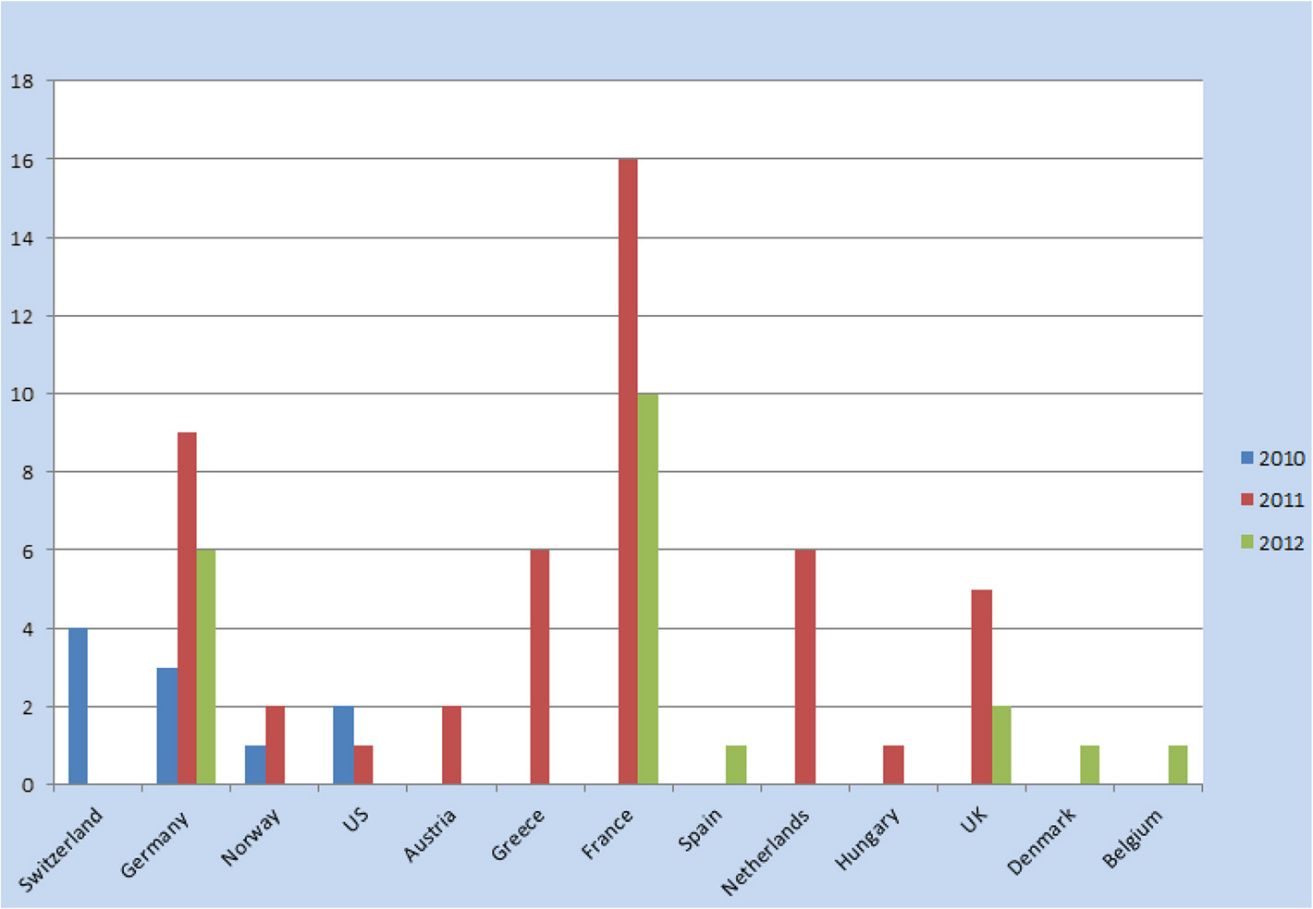
**Pediatric patients enrolled annually 2010-2012.** The number of patients enrolled each year between 2010 and 2012 from each enrolling country.

In the first year of recruitment only four countries were enrolling patients, and a very low recruitment of paediatric patients had been achieved (n = 10). The Steering Committee reviewed the registry strategy and recommendations were made to enhance recruitment. Identification of national professional CAPS opinion leaders and individual centers with relatively active CAPS programs was adopted as a potentially successful strategy for reaching target numbers. The automated system was well accepted and centers reported that enrolment and follow-up became progressively easier with time and practice. Thus, the Steering Committee recommended two modifications of the protocol. The first was an extension of enrolment in designated sites for a second year. This was deemed feasible and cost-effective, since once trained on the automated system, practitioners reported that they found enrolment of subsequent patients in their practices much less burdensome. The analysis plan was modified to accommodate such continuing enrolment and the resulting eventual truncation of data for those with less time in the Registry. The second modification proposed was based on the first year’s experience of very low recruitment of paediatric patients. Thus, it was decided to direct further efforts towards recruitment at the new Registry sites with a focus on paediatric patients. Figure 3 shows the annual enrolment of paediatric patients, per country. At the end of the second year, recruitment targets for adults were well met and the targeted sample size was achieved. The focus on further enrolment will be therefore limited to paediatric patients.

## Discussion and conclusion

As with all very rare diseases, the clinical development program with canakinumab included a very limited number of patients for a necessarily brief period of follow-up; therefore, the post-approval period was deemed a critical phase in which to gather more knowledge regarding the short- and long-term safety, effectiveness and treatment patterns associated with use of the product by clinicians and patients. Non-intrusive, wholly observational registry approaches are appropriate for such situations. The complexity of patients’ co-medications and co-morbidities can well be captured by an observational/registry study [9]. However, the rarity of the condition poses some specific challenges because any one practice, even specialty practices, will likely only have a few patients, so that the treating physician needs to have clear guidance about which patients to include in the registry, and periodic reminders to keep the participation and data capture to acceptable levels. Methods for this registry were developed in accordance with the STROBE guidelines [7] and the Registries User’s Guide, which importantly sets up criteria and recommendations supporting the planning, design and implementation of registries including considerations on data collection and quality assurance [8].

For the CAPS registry, a robust system for data collection was adopted. The CAPS registry approach features global reach, often with national coordinators/advocates; intensive collaboration with/ownership by opinion leaders/referral centres; targeted local and area programs of disease and registry awareness; and innovative low effort methods (web-based) of data collection.

An additional challenge regarding recruitment of these patients was due to the fact that these conditions can not only be diagnosed by paediatricians/ paediatric rheumatologists, but also by specialists caring for adult patients within and outside of rheumatology (e.g. dermatologists, immunologists and others). Thus, the referral centers were encouraged to extend the net of participating centers as widely as possible. This allowed capturing a more detailed natural history of the disease and understanding the gap between onset of disease and diagnosis, and treatment.

Electronic data collection was field tested for ease of introduction and use. It was recognized that adopting a simple, user-friendly, and robust electronic data capture system can facilitate the data collection process. As the experience with the electronic data collection method progressed, the treating physicians and their staffs reported that they found enrolment of subsequent patients in their practices much less burdensome. One of the challenges of a data collection system is striking a balance between the necessary data to collect and additional information that may be deemed useful for a more in depth knowledge of the natural history of the disease but may be perceived as burdensome by the physician and lead to increase in missing data. Through periodic analyses of the data, to coincide with the periodic safety update reports, the team has gathered information regarding completeness of data and initiated the necessary measures to reduce missing data and improve data quality, including monitoring visits to the sites, and implementing continuous training of the participating physicians.

The continuous engagement of participating physicians and centers also ensures that the follow-up of these patients is as complete as possible and that safety information and data related to the progression of the disease can be reported in the registry. Continuous engagement of the team ensures the patients’ follow-up is monitored and the relatively small proportion of patients who discontinued the study to date is testimony to the appropriate monitoring measures in place.

Product registries, by definition, focus on patients treated with a particular medicinal product; this kind of registry is therefore not inclusive of all patients within a disease category, since it does not cover those patients who have been diagnosed with the condition but follow a different treatment regimen. This is one of the limitations of the study that prevents direct comparison of patients treated with canakinumab to those treated with other medications. The Registry can also not be considered a complete source of data regarding the “natural history of disease”, since, by definition, it follows only patients who have received one or more interventions aimed at treating their condition, and only patients with disease progression and treatment experience warranting initiation of a new therapy enter follow-up. However, even such patients may provide rich history of untreated disease at the time of enrolment. Further, the voluntary nature of enrolment introduces other possible selection biases. Despite these limitations product registries can be important resources, if the representativeness and relevant characteristics of the exposed population are carefully considered, especially in the immediate post-approval setting, and specifically for safety information. In this respect, orphan drug registries may also become relevant in adaptive licensing approaches in which traditional drug licensing (which is based on binary decisions), is transformed into a stepwise learning process under conditions of acknowledged uncertainty, with iterative phases of data gathering and regulatory evaluation [10].

The convergence of short exposures to chronic treatment requires creative approaches to observational methodology, harnessing registry strategies with novel approaches to recruitment of reporting Health Care Providers (HCPs) and monitoring of their patients.

From a strategic point of view, a registry, as part of a post-approval program of safety monitoring, has the following advantages compared to a randomized clinical trial (RCT). A registry allows collection of information about practice in routine clinical care. All patients receiving the treatment of interest can be included (without artificial exclusions); there is no dictated treatment regimen. Moreover, a registry is conducted in “real world patients” (who typically have higher baseline risk than patients enrolled in RCTs) and therefore has good external validity and finally, a registry allows for prospective data collection and for a longer term follow-up than a clinical trial.

A cheaper alternative to a registry could be represented by data collection from some form of pre-existing database, e.g. electronic medical records or administrative data (or even medical records abstraction). Such methods have been used in other efforts to monitor rare diseases. However the standard of care may differ between different countries, and there is no guarantee that the data collection routinely in place for such a disease would be comparable between different countries, or even between different physicians in the same country.

From a methodological point of view, rare diseases pose significant challenges for epidemiologic follow-up study. EDC and a central office for technical assistance as features of this Registry can help to overcome resistance to participation and facilitate comprehensive data collection. The recruitment of experienced clinicians in each country to assist in engaging their colleagues in participation in the registry provides access to the network of colleagues with shared interest in rare disorders.

The Advisory Board also serves as the Publications Committee for the Registry, to ensure that information about the Registry and its emerging findings can be suitably analyzed and independently submitted for publication. The Committee also invites the Registry investigators to submit suggestions for publication and participate in such activities, reserving, however, all rights of access to the overall data base to prior review and approval by the Publications Committee in accordance with the European Network of Centres for Pharmacoepidemiology and Pharmacovigilance (ENCePP) code of conduct for pharmacoepidemiological and pharmacovigilance studies [11].

An international registry can fill the knowledge gap both by shedding light on the natural history of the disease and by monitoring the response to a new treatment including detection and quantification of potential adverse experiences and monitoring of effectiveness outcomes. However, for rare diseases, special efforts are needed, including identification of local opinion leaders and electronic data capture.

## Abbreviations

CAPS: Cryopyrin-associated periodic syndromes; FCAS: Familial cold autoinflammatory syndrome; MWS: Muckle-wells syndrome; NOMID: Neonatal onset multisystem inflammatory disease; CIAS1: Cold-induced auto-inflammatory syndrome 1; NLRP3: Nod-like receptor protein 3; CINCA: Chronic infantile neurologic cutaneous articular; SJIA: Systemic juvenile idiopathic arthritis; FMF: Familial mediterranean fever.

## Competing interests

HT, TV and PNH are member of The Ilaris^®^ Registry Study Steering Committee. PP and BR are employees of Novartis. DK was an employee of Novartis and holds stocks of Novartis. HH is a consultant for Novartis, Sobi, Regeneron pharmaceuticals and a speaker and advisory board member for Novartis and Sobi. JKD has received travel reimbursements, honoraria for speeches and research grant support from Novartis. UAW receives reimbursement from Novartis for his work with the Ilaris registry.

## Authors’ contributions

All Authors contributed in the design of the study. BR and DK participated in the coordination of the study. HT and PP drafted the manuscript. DK, BR, PNH, HH, JKD, TP, UAW made substantial contribution in the interpretation of the results and critically revised the manuscript. All authors read and approved the final manuscript.

## Authors’ information

HT is Board-certified in Public Health and Preventive Medicine for 40 years. He directed the program in drug safety surveillance and epidemiology for Wellcome (latterly GSK) for 15 years. He is founding member and lifetime (honorary) Fellow of the American Academy of Pharmaceutical Physicians (latterly APCR) and the International Society for Pharmacoepidemiology (ISPE), Co-Editor of “Pharmacoepidemiology: An Introduction” and “Pharmacoepidemiology and Therapeutic Risk Management” (Harvey Whitney Press, Cincinnati, OH). He is Consulting Epidemiologist and author for multiple multinational studies. DK is Head of Medical Affairs at Spectrum Pharmaceuticals. Previously, Global Program Medical Director at Novartis. PP is a member of International Society for Pharmacoepidemiology. BR is a clinical scientist at Novartis and has experience in conducting clinical trials including registries. PNH is head of the UK National Amyloidosis Centre and the UK CAPS Treatment Service at Royal Free Hospital, London. He pioneered interleukin-1 blockade as a treatment for CAPS in 2002, and was Principal Investigator on the pivotal clinical trials of canakinumab for the treatment of CAPS. HH is MD with training in pediatrics and allergy and immunology and Expert in autoinflammatory diseases. He is Professor of Pediatrics and Medicine, University of California at San Diego and Rady Childrens Hospital San Diego and Secretary of International society of systemic autoinflammatory diseaeses JKD is a Board-certified Pediatrician and a Board-certified Pediatric Rheumatologist. She is Head of the Division of Pediatric Rheumatology; Department of Pediatrics, University Hospital Tuebingen, Germany, Head of the autoinflammation reference center Tuebingen, Germany. She is a member of American college of Rheumatology, Pediatric Rheumatology European Society, International Society of Systemic Auto-Inflammatory Diseases Deutsche Gesellschaft für Rheumatologie Gesellschaft für Kinder- und Jugendrheumatologie. TV is Board-certified in Internal Medicine and Infectious Diseases. He heads the Division of Infectious Diseases and Center for Experimental and Molecular Medicine at Academic Medical Center and is Professor of Medicine at the University of Amsterdam, the Netherlands. He is Chair of AMC Research Council & Department of Research Support and Internation Sepsis Forum. He is a member of advisory boards and steering committees of several pharmaceutical companies, Data Safety Monitoring Boards and Clinical Evaluation Committees of several trials on sepsis, pneumonia and rheumatoid arthritis UAW is Board-certified internist, rheumatologist, infectious disease specialist and allergologist. He is appointed as professor and Head of the outpatient rheumatology service at Basel University. He has authored more than 140 peer-reviewed publications and 30 book chapters. He has a research laboratory focusing on the role of mitochondria in degenerative, metabolic and inflammatory diseases.
